# Understanding and Treating Persecutory Delusions

**DOI:** 10.1093/schbul/sbae012

**Published:** 2024-02-03

**Authors:** Daniel Freeman

**Affiliations:** Department of Experimental Psychology, University of Oxford, Oxford, UK; Oxford Health NHS Foundation Trust, Oxford, UK

*“I’ve looked at clouds from both sides now.” Joni Mitchell* ^1^

When I began my journey as a clinical psychologist, I knew nothing about persecutory delusions. At least, little that was correct. The prevailing psychiatric view in the 1990s was easily comprehensible though. Paranoia, it was believed, occurred only in people with a mental health problem. Persecutory delusions were merely a symptom of severe conditions such as schizophrenia. One needed simply to treat the schizophrenia (principally by medication) and the paranoia would disappear. Nothing could be achieved by talking with patients about their fears—indeed, talking could make things worse. Except as a diagnostic indicator, the content of the delusion was irrelevant.

The first time I talked with a patient in detail about their persecutory delusions made it clear that these tenets were probably wrong. The prevalence, causes, and ways to overcome paranoia were likely a lot more complicated, a lot more plausible, and far more interesting than the consensus would have one believe. What follows are some of the lessons I’ve since learned from clinical research and practice.

1. Persecutory delusions are the severe end of a paranoia dimension in the general population

Every day we must decide whether to trust other people. It’s not always easy: reading other people’s intentions is a tricky business. When our assessment of those intentions is skewed towards the negative, that is paranoia. Specifically, paranoia is thinking that others are deliberately trying to harm you when they are not. Many people have a few paranoid thoughts, and a few people have many. In my view, people with troubling persecutory delusions are at the extreme end of a continuum, and these delusions are not “qualitatively different” from less severe paranoid experiences. Their causes are the same. What pushes a person further along the continuum is the number and severity of those causal factors (a dose–response relationship).^2,3^ This is a radical reconceptualization of paranoia that moves far beyond seeing it simply as a symptom of illness. It is not an uncontested view.^4^ A continuum perspective on paranoia is consistent with views of other problems such as anxiety and depression.^5^ And it makes paranoia—in clinical and non-clinical populations—worthy of study in its own right.

2. Paranoia as a battle

Persecutory delusions are inaccurate threat beliefs. An individual who perceives others as trying to harm them can easily come to see life as inherently conflictual. As a patient told me: “My life was a constant battle. I felt far too paranoid to participate in so many events and activities. I used to keep all my curtains shut and had all doors locked . . . I just felt that there was a ticking time bomb till I got attacked or gave up myself.”^6^ It is a battle in which patients typically feel vulnerable to harm—they start from a weakened position. As in any battle, the person tries to defend themselves. But these defences—eg, avoiding activities, shutting curtains, avoiding eye contact—prevent the receipt and processing of disconfirmatory evidence. In other words, what the defences end up protecting isn’t the individual but rather the false ideas. In a world of ticking time bombs, paranoia can feel strategic. “Survival,” wrote John le Carré,^7^ “is an infinite capacity for suspicion.”

3. Be open to the evidence for the delusion

Before every appointment with a patient, I remind myself of the words of Brendan Maher^8^: “the delusional belief is not being held ‘in the face of evidence normally sufficient to destroy it,’ but is being held because of evidence powerful enough to support it.” If we truly want to effect change, we must understand what that supporting evidence is. In part, it may be the long shadow of negative events from the past, which have taught the person that they are vulnerable and others hostile. In the moment that the thoughts are experienced, heightened anxiety often drives misinterpretation. A range of other subtle perceptual anomalies, including dissociation,^9^ may also signal that there are reasons to be fearful. And then there are verbal auditory hallucinations. Voices are often an important informational source for persecutory delusions, undermining the person’s self-esteem so that they feel vulnerable and/or directly warning of potential attacks.

4. There are real dangers

We need to be clear that real dangers exist, that people do bad things to others, and that safety can seldom be guaranteed. Recognizing these facts is not paranoia. Paranoia can be seen as an example of risk assessment gone awry. Moreover, the defences adopted in the face of perceived threat tend to bring additional problems.

5. Causation is multi-factorial

Multiple factors are needed to explain paranoia, and these may vary in the individual instance. Each cause is likely an “inus condition”^10^—“an insufficient but non-redundant part of an unnecessary but sufficient condition.” A single cause therefore only increases the probability of a delusion occurring. Greater exposure to environmental risks lessens the need for genetic risk for paranoia to occur.^11^ In a recent study, we found that thirteen factors were required to explain two-thirds of the variance across the population in paranoia.^12^ In reality, paranoia is likely to emerge from a complex network of interacting factors,^13,14^ many of which will be shared with other common mental health problems.

6. Develop and use better measures

Increasingly, I find that each advance in the theory or treatment of paranoia requires the development of better assessments. This is time well spent. Precise measurement is a powerful means to drive understanding, identify treatment targets, and evaluate outcomes. Regarding the nature of that measurement, however strong the desire to identify biomarkers the best source of information will be patient self-report. Of course, a few patients sometimes do not report accurately. But this is no reason to dismiss the testimony of the overwhelming majority. A far bigger problem is that existing measures are frequently neither patient-centered, psychometrically robust, nor indeed even used in services. When it comes to patient benefit, the single most important innovation would be for services to repeatedly assess patients on appropriate well-developed measures and use the results to guide, monitor, and, when needed, alter the treatment provision.

7. The antidote is the learning of safety

Recovery from paranoia means learning that the world is safe enough now. This learning must be direct—involving the formation of new memories—and it must be sufficiently practiced to ensure that it becomes the new dominant narrative. There are however a number of psychological processes that can prevent such learning even when no harm is happening. For example, anxious worrying, feelings of vulnerability, and the use of defences can all make safe situations feel dangerous. Therefore a person needs to be in the right psychological state to make use of exposure to safe situations. Recovery is about learning where there is genuine safety, or at least very low risk, in the person’s life.

8. Do not underestimate patients

There are reasons for optimism in the treatment of severe paranoia. The Feeling Safe programme, developed over two decades, leads to recovery in delusions for half of the patients who have not responded to anti-psychotic medication.^15^ Another quarter of patients get the benefit. However, we cannot predict who will respond. Even patients with the severest psychosis presentations can make gains. So, we should not underestimate the ability of any patient to get back to their chosen activities. I have also noticed that when technological innovation—eg, smartphones or virtual reality—is suggested some mental health professionals are initially wary. Yet in my experience, provided there is a good rationale behind the use of technology, and that rationale is clearly explained, patients are delighted to be given access. Finally, there are of course patients for whom current treatment offerings do not work or could be improved. In that regard, we shouldn’t underestimate the insights patients can offer into the causes of delusions. These insights can be used to develop better interventions—if we take the time to listen.

The dominant paradigm in psychosis research is trying to explain diagnoses such as schizophrenia or, at best, clusters of symptoms within the diagnosis. Yet this approach seems to me inherently flawed. It is obvious that experiences such as paranoia, grandiosity, hearing voices, thought disorder, anhedonia, and so forth are quite different. There will be a degree of shared causation, for sure, but these experiences require individual study in order to develop treatments with large benefits for patients. The example of paranoia shows how, even in a short period of time, a focus on a specific experience can lead to a radical shift in understanding. It demonstrates how much therapeutic progress can be made. And it reveals how, despite—or perhaps because of—the specificity of its focus, wider implications for human behavior can emerge. That said, there is still much more to learn about paranoia and much work remaining to translate and implement these advances into mental health services.
